# The Research Domain Criteria (RDoC) domains positive valence system, negative valence system, cognitive systems, and social processes and their relationship with stress, anxiety, and depressive symptoms in a university student sample

**DOI:** 10.3389/fpsyt.2026.1674802

**Published:** 2026-03-05

**Authors:** Veronika Esipova, Mira Tschorn, Sarah J. Böttger, Julia Seiffert, Bernd Förstner, Timm Seegert, Michael A. Rapp, Kristin Koller-Schlaud

**Affiliations:** 1 Social and Preventive Medicine, Department of Sports and Health Sciences, University of Potsdam, Potsdam, Germany; 2 German Center for Mental Health (DZPG), partner site Berlin/Potsdam, Berlin, Potsdam, Germany; 3 Academic Sports Center, Student Health Management, University of Potsdam, Potsdam, Germany; 4 Department of Psychiatry, Psychotherapy and Psychosomatics, University Hospital Ruppin-Brandenburg, Brandenburg Medical School, Neuruppin, Germany

**Keywords:** cognitive systems, faculty, negative valence system, positive valence system, RDoC, research domain criteria, social processes, student mental health

## Abstract

**Introduction:**

Mental health difficulties are highly prevalent among university students. The Research Domain Criteria (RDoC) framework seeks to improve diagnosis, treatment, and prevention by providing a comprehensive framework for understanding mental health and illness. We report the prevalence of stress, anxiety, and depressive symptoms in a student sample and investigate their relationship with the RDoC domains positive valence system (PVS), negative valence system (NVS), cognitive systems (CS), and social processes (SP). Differences by faculty affiliation are also explored.

**Methods:**

184 university students (mean age 24.5 ± 5.9 years, 73.4% female) participated in an online survey. RDoC domain scores were determined using a confirmatory factor analysis. Stress, anxiety, and depressive symptoms were assessed using the Depression Anxiety Stress Scale (DASS-21). Multiple linear regressions (MLR) were conducted to examine RDoC domain factor scores as predictors of stress, anxiety, and depressive symptoms. Additional MLR analyses examined faculty affiliation as a predictor.

**Results:**

30% of the participants (stress 28.8%, anxiety 31.0%, depressive symptoms 30.4%) showed an elevated symptom burden. NVS- and CS-scores were positively associated with stress (*b* = 3.417, *p* < 0.001, *b* = 1.412, *p* = 0.001), anxiety (*b* = 2.735, *p* < 0.001, *b* = 0.994, *p* = 0.003), and depressive symptoms (*b* = 1.519, *p* = 0.004, *b* = 1.000, *p* = 0.012). PVS- and SP-scores were negatively associated with depressive symptoms (*b* = -1.306, *p* = 0.006, *b* = -2.050, *p* = 0.001). No differences by faculty affiliation were found.

**Conclusions:**

Our findings demonstrate a notable mental health burden in university students. We found associations between RDoC domains and all three mental health outcomes. Depressive symptoms were associated with all assessed RDoC domains. Anxiety and stress were linked to NVS- and CS-scores. Mental burden was therefore predominantly associated with heightened negative affect, anxiety, and subjectively perceived impaired cognitive performance. Depressive symptom burden was additionally associated with reduced positive affect and impaired social behavior and interaction. No faculty-specific burden was observed. The study contributes to understanding university students’ mental health burden and represents a further step in conceptualizing stress, anxiety, and depressive symptoms based on the RDoC framework in a non-clinical sample.

## 1 Introduction

One tenth of university students in Germany rated their overall subjective health as fair or poor in 2023, while about two thirds rated their health as good or very good (1). Even though this reflects a large proportion with a positive rating, the overall rating of university students is still significantly lower than that of an age-matched representative sample (2). Moreover, compared to 2015 when only 3% of students reported negative health ratings, the proportion since then has more than tripled (1). The differences in health ratings between 2015 and 2023 may in part reflect the impact of the COVID-19 pandemic, during which university students experienced university closures, a shift to online learning, and prolonged social restrictions, which likely contributed to increased isolation, disruptions in lifestyle, and financial stress (3, 4), affecting overall health perceptions and well-being. Consistent with this, studies reported declines in student mental health during the pandemic, including increases in depressive symptoms and impairments in overall well-being (4–6). Overall, mental health problems are prevalent among university students and particularly stress, anxiety, and depressive symptoms have a notable negative impact in this population (1, 7).

Stress, for example caused by academic pressures or the burden of having a side job, affects around half of students, with a quarter of them experiencing very high levels of stress (1, 7, 8). Prolonged stress also increases the risk of developing symptoms of anxiety disorders such as generalized anxiety disorder, which was reported by 17.4% of university students in a study in Germany (8). Furthermore, stress has been linked to depressive symptoms and 15.6% of university students reported in 2017 that they were affected by depressive symptoms (8). However, only 5% of those reporting symptoms received medication (1). Depressive symptoms have been associated with life dissatisfaction, social isolation, insomnia (e.g., (9, 10)), poor academic performance (11), and increased health risk behaviors (12) in university students. Stress has also been shown to decrease academic performance in university students (13) and increase the risk of university dropout (14) in addition to negatively affecting social and general well-being (15, 16), quality of life (17), and professional opportunities post graduation (18). Since stress is a known risk factor for the development of mental illnesses (e.g., (19); for a review, see (20)), long term consequences may also include financial expenses for treatment in addition to broader societal and economic costs (for a review, see (21, 22)). Indeed, the World Health Organization World Mental Health Surveys (23) revealed that one-fifth (20.3%) of college students had a 12-month prevalence of an anxiety, mood, behavioral, or substance use disorder, however, only 16.4% of students within a diagnosis within the past 12 month received any healthcare treatment during that time, making both prevention and treatment a crucial topic in this population.

Study conditions such as high study demands, limited time flexibility and a lack of social support have been shown to influence perceived psychological stress (24). Regarding differences among academic disciplines, Grützmacher et al. (8) showed that students of linguistic and cultural studies were disproportionately affected, exhibiting higher levels of depressive and anxiety symptoms. Furthermore, only 77.6% reported good or very good self-rated health, compared with significantly higher percentages in law and economics (84.5%), engineering (84.9%), and medicine and health sciences (85.5%). A survey of students at Freie Universität Berlin (25) revealed that students of veterinary medicine (62.7%), law (57.3%), and physics (56.4%) were particularly affected by stress. The causes for these differences however, remain unclear. It is conceivable that these differences reflect variations in study conditions, such as academic structures, workload, and assessment formats across fields, as study conditions have been shown to influence psychological stress (24). However, existing findings do not allow firm conclusions. For example, Olson et al. (26) report in their study on stress and student burnout that their findings are not fully consistent with a study-demands resources model and suggest that future research should investigate whether differences between faculties are causal and whether they arise from structural or personal factors.

Prevention, treatment, and research around mental disorders has been mostly centered around established diagnostic systems, namely the International Statistical Classification of Diseases and Related Health Problems (ICD) and the Diagnostic and Statistical Manual of Mental Disorders (DSM). Despite their wide use and recognized merit, these classification systems meet limitations associated with their categorial approaches, which has recently prompted calls for more individualized approaches (27, 28). Underlining disadvantages of categorical systems, the frequent co-occurrence of diagnostic categories has been pointed out as a concern (28). The Research Domain Criteria (RDoC) framework, initiated and developed by the National Institute of Mental Health in 2009 (29), addresses these concerns by providing a comprehensive and structured framework to guide psychobiological research efforts on mental illness and health using neurological circuit-based behavioral dimensions that cut across traditional diagnostic categories (30).

Allowing for a deeper understanding of the continuum from mental health to mental disorder in its various degrees of severity, the RDoC approach focuses on understanding mental states by integrating biological, physiological, and behavioral approaches to improve diagnosis, treatment, and prevention (28–30). The heuristic framework is currently organized into six different domains of basic neural function and human behavior, taking into account developmental and environmental influences.

The six domains include the negative valence system (NVS), the positive valence system (PVS), cognitive systems (CS), social processes (SP), arousal and regulatory systems, and sensorimotor systems. All domains are further subdivided into hierarchically arranged constructs. The PVS-domain comprises the reaction to and processing of positive stimuli and reward (31, 32). This domain is divided into the three constructs responsiveness to reward, reward learning, and evaluation of reward (31). The NVS-domain encompasses the reaction to unpleasant or negative stimuli and situations, comprising the constructs acute threat (fear), potential threat (anxiety), persistent threat, loss, and frustrative non-reward (31). The CS-domain encompasses a range of cognitive processes, including the six constructs attention, perception, declarative memory, language, cognitive control, and working memory (31). The SP-domain encompasses human social behavior and consists of the four constructs affiliation and attachment, social communication, perception and understanding of one’s own self as well as perception and understanding of other people. The units of analysis in the RDoC matrix include the genetic, molecular and cellular level, as well as investigations based on brain circuits, physiology, behavior, self-report, and paradigms (31).

While individual RDoC domains, most notably the negative and positive valence systems, have been linked to depression or anxiety, empirical evidence remains limited for associations across multiple dimensions of psychological burden, such as stress, anxiety, and depressive symptoms, and across multiple RDoC domains within the same sample. In particular, domains beyond NVS and PVS have been less frequently examined, particularly in non-clinical samples. However, previous findings and theoretical frameworks can guide assumptions about these associations. In their review on the relationship between stress and the reward system (PVS-domain) with a focus on adolescence and emerging adulthood, Auerbach et al. (33) include a study by Berenbaum and Connelly (34) that found a negative effect of stress on pleasure in college students following an examination phase. These findings point to a specific premorbid vulnerability in reward processing, particularly affecting responsiveness to and processing of rewarding stimuli. The direction of this relationship however, is unclear, as inhibited reward processes can be both cause and result of stress (33). Chronic stress can be conceptualized within the NVS-construct of sustained threat, hence, chronic stress can be assumed to be linked to high levels of NVS expression (35, 36). Regarding potential links to the CS-domain, it is to note that central brain circuits are involved in stress reactions, which have a relevant influence on cognitive functioning, such as memory performance, attention, and decision-making (for a review, see (37)). Liston et al. (38) showed an impairment of cognitive systems and processes, such as slowed down attention due to prolonged stress. The SP-domain is closely linked to stress: Acute stress inhibits dopamine regulation, reducing motivation for social interaction, attachment, and responsiveness to positive stimuli. Prolonged, uncontrollable stress can lead to neural deterioration in regions responsible for social behavior, resulting in impaired emotion regulation, increased impulsivity, and poor situational judgment (39).

In regard to anxiety, Förstner et al. (40) found that higher scores on the PVS-domain were linked to lower severity in anxiety disorders. They also identified a positive association between increased anxiety and the NVS. Cognitive dysfunctions in anxiety disorders have been repeatedly reported (for a review, see (41)), however, the direction of the relationship between cognitive impairments and anxiety disorders, is unclear and the specific nature of cognitive impairments may depend on the type of anxiety (41, 42). In anxiety disorders, Förstner et al. (40) found that poorer social-process functioning (e.g., lower social skills, greater interpersonal sensitivity/hostility, and more social anhedonia) was associated with higher symptom severity, though this inverse association was weaker than in major depression (40). People with generalized anxiety specifically show deficits in the construct of perception and understanding of self (43).

In regard to depressive symptoms, Auerbach et al. (33) highlight that an impairment of reward processes due to high stress load is associated with depressive symptoms. Reduced PVS functioning has been linked to depression across different units of analyses (44). Förstner et al. (40) found that greater depression severity correlated to lower PVS functioning and that this link was stronger in depression than in anxiety disorders. Studies report a positive correlation between the severity of diagnosed depression and NVS-constructs (40, 45). Moreover, depression is also associated with impairments in cognitive functions including attention and working memory, with greater cognitive impairments linked to more severe depressive symptoms (42, 46). Finally, depression can negatively affect social behavior, such as disrupting relationship and attachment behavior and increasing the sensitivity for perceived rejection, with the severity of these social impairments more closely related to depression than anxiety disorders (40, 46).

The aim of this study was to investigate the relationship between the PVS-, NVS-, CS-, and SP-domains and the mental burden of stress, anxiety, and depressive symptoms in a university student sample. Since previous studies have reported differences in symptom burden between students of different fields of study, the relationship between faculty affiliation (group of related sciences or scientific fields as a comprehensive department at a university (47, 48)) and the severity of mental burden among students of different fields at the University of Potsdam (Universität Potsdam, UP) was also investigated.

The present study addresses the following research questions: What is the relationship between the scores of the PVS-, NVS-, CS-, and SP-domains and the current levels of stress, anxiety, and depressive symptoms among students at the University of Potsdam? Are there significant differences in stress, anxiety, and depressive symptom burden across students from different faculties in the present university student sample?

## 2 Materials and methods

### 2.1 Study design

The data used for the present analyses originate from the survey “MDS - Validation of a Minimum Data Set for the transnosological assessment of mental health” (MDS-Valid) by the “German Center for Mental Health” (DZPG). A cross-sectional anonymous online survey was conducted in the DZPG-affiliated healthcare centers as well as at the University of Potsdam (Germany). Data collection at the health care centers took place between January 31^st^ and April 29^th^, 2024. Data collection for the student sample took place at the University of Potsdam from December 11^th^, 2023 to February 5^th^, 2024 on the survey system Umfragen.UP (based on SoSci Survey), which was accessed through a link or QR code. The student health management team promoted the survey using emails, flyers, posters, and personal contact within the university context at all three UP campuses. The study was approved by the local ethics committees of the University of Potsdam (vote 06/2016, amendment 59/2023 for health care centers and 100/2023 for the student sample) and was conducted in accordance with the Declaration of Helsinki. Data from the online survey of the affiliated health care centers was used for conducting a confirmatory factor analysis (see 2.2. and **Supplementary Material S1**). The analyses for the main research questions were conducted on the university student sample only.

### 2.2 Estimation and interpretation of domain scores

The latent RDoC domain structure was determined using a confirmatory factor analysis (CFA). The CFA was carried out in alignment with the method described by Förstner et al. (13). For the calculation of the domain factor scores, data of the UP student sample (n = 249, 41.8%) and data collected at the DZPG-affiliated health care centers (n = 347, 58.2%) were combined to yield a total sample of 596. The inclusion of the health care center data increased both sample size and heterogeneity, supporting a more robust estimation of the latent RDoC domains, while the main analyses were restricted to the student sample.

The CFA was carried out using the maximum likelihood method. The Full Information Maximum Likelihood (FIML) method was applied to address missing data. The comparative fit index for the model of the four domains of interest resulted in a value of CFI = 0.91 and thus indicated a sufficient fit for the data collected. Factor scores were derived from the CFA model with z-standardized indicators, resulting in scores approximating standardized factor scores (mean (M) = 0, standard deviation (SD) = 1). The Shapiro-Wilk test was applied to test for normal distribution of data. Certain items were reversed for easier interpretation of the polarity of the domain scores. The CFA was carried out in R version 4.2.2 with the package lavaan version 0.6–12 and RStudio version 1.3.1073. For detailed information on the analysis see **Supplementary Material S1**. The PVS-domain score is based on items on positive affect (PANAS: active, interested, excited, determined, (49)). The NVS-domain score is based on items on acute threat (subjective units of distress, (50)), anxiousness, phobic anxiety, and negative affect (PANAS: irritable, ashamed, (49)). The CS-domain score is based on items on problems with executive functioning, such as flexibility, planning, and working memory functioning. The SP-domain score is based on items on social support, experiencing social exclusion, interpersonal sensitivity, social anhedonia, and maintaining friendships. In general, higher RDoC domain factor scores indicate higher levels of functioning. However, for the CS-domain in the present study, the factor score derived from the CFA differs in its interpretation from the RDoC definition, as higher CS factor scores reflect greater impairment in executive functioning rather than higher functioning. For further descriptions of the assessments used for the CFA see **Supplementary Material S1**.

### 2.3 Depression Anxiety Stress Scale

The short version of the Depression Anxiety Stress Scale (DASS-21; (51)) was used to assess the degree of mental burden related to stress, anxiety, and depressive symptoms. The DASS-21 consists of a set of three self-report scales containing 7 items each. Statements are rated on a four-point Likert scale ranging from “0” (did not apply) to “3” (applies most of the time), referring to the past 7 days. A score of 10 or higher indicates a significantly increased burden of depressive or stress symptoms. On the anxiety subscale a score of 6 or higher indicates an increased level of anxiety symptoms. Good psychometric properties including high reliability (Cronbach’s alpha between .78-.91), and strong validity have been demonstrated (51).

### 2.4 Sample

A total of 249 UP students participated in the survey, with 73.9% (N = 184) providing sufficiently complete data to be included in the analysis based on data sufficiency. Of the participants, 73.4% (n = 135) identified as female, 24.5% (n = 45) as male, 0.5% (n = 1) as diverse, and 1.6% (n = 3) did not specify their sex. The average age was 24.5 ± 5.9 years, with male students reporting slightly older age (25.5 ± 7.7) than female students (24.1 ± 5.2). Most students (53.8%, n = 99) were pursuing a bachelor’s degree, 35.9% (n = 66) a master’s degree, 5.4% (n = 10) a state examination, and 4.9% (n = 9) selected “other” (**Table 1**).

**Table 1:** Severity of stress symptoms, anxiety symptoms, and depressive symptoms on the DASS-21 and the domain scores (PVS-score, NVS-score, CS-score, SP-score) across the total sample and by faculty affiliation.

Group	N (%)	DASS-stress (*M* ± *SD*)	DASS-anxiety (*M* ± *SD*)	DASS-depression (*M* ± *SD*)	PVS-score (*M* ± *SD*)	NVS-score (*M* ± *SD*)	CS-score (*M* ± *SD*)	SP-score (*M* ± *SD*)
Total sample	184 (100.0)	7.0 ± 4.6	4.3 ± 3.9	7.0 ± 5.3	0.17 ± 0.85	-0.15 ± 0.84	-0.14 ± 0.86	0.17 ± 0.83
Law Faculty	11 (6.0)	6.5 ± 3.7	2.8 ± 1.5	7.0 ± 4.7	0.43 ± 0.94	-0.29 ± 0.58	-0.23 ± 0.93	0.48 ± 0.82
Faculty of Arts (interdisciplinary cultural studies)	25 (13.5)	6.3 ± 4.7	4.2 ± 3.4	7.1 ± 4.5	0.28 ± 0.74	-0.10 ± 0.67	-0.18 ± 0.77	0.18 ± 0.57
Faculty of Human Sciences	53 (28.8)	7.0 ± 4.3	3.9 ± 3.7	6.2 ± 4.9	0.30 ± 0.83	-0.23 ± 0.80	-0.20 ± 0.82	0.29 ± 0.83
Faculty of Economics and Social Science	25 (13.6)	6.8 ± 5.8	4.6 ± 4.9	6.3 ± 5.9	0.06 ± 1.04	-0.23 ± 0.96	-0.31 ± 0.88	0.17± 0.98
Faculty of Science (Mathematics and Natural Sciences)	57 (31.0)	7.8 ± 4.7	4.9 ± 4.1	8.0 ± 5.6	-0.03 ± 0.82	0.02 ± 0.90	0.08 ± 0.93	-0.00 ± 0.87
Digital Engineering Faculty	13 (7.1)	5.4 ± 3.8	4.1 ± 4.2	7.9 ± 6.2	0.26 ± 0.67	-0.42 ± 0.88	-0.33 ± 0.69	0.15 ± 0.69

*DASS-21*, Depression Anxiety Stress Scale; *DASS-stress*, Depression Anxiety Stress Scale subscale stress; *DASS-anxiety*, Depression Anxiety Stress Scale subscale anxiety; *DASS-depression*, Depression Anxiety Stress Scale subscale depressive symptoms; *PVS-score*, domain score positive valence system; *NVS-score*, domain score negative valence system; *CS-score*, domain score cognitive systems; *SP-score*, domain score social processes; *M*, mean; *SD*, standard deviation. DASS-stress: 2 missing, DASS-anxiety: 2 missing, DASS-depression: 1 missing. For the statistical analysis of group differences regarding faculty affiliation, a single-factor analysis of variance according to Welch (*p* ≤ 0.05) was carried out. There were no significant differences between the faculties regarding DASS-21 (*p* > 0.05).

### 2.5 Statistical analysis

All statistical analyses were performed using IBM SPSS Statistics version 28.0 (52). Two-tailed tests were applied, and results are reported as statistically significant at a level of *p* ≤ 0.05.

To analyze the relationship between domain factor scores as well as faculty affiliation and outcome variables (DASS-21), six multiple linear regressions (MLR) were performed with sex as a covariate. Prior to conducting the analysis, we assessed key statistical assumptions: normal distribution of residuals (Shapiro-Wilk test), homoscedasticity (Breusch-Pagan test), absence of autocorrelation (Durbin-Watson statistic), linearity (partial regression plots for metric predictors), and low multicollinearity (variance inflation factor (VIF) and tolerance). Outliers (n = 7) defined as values exceeding ± 3 SD, were excluded following a plausibility check (domain scores and stress = 2; domain scores and anxiety = 2; domain scores and depressive symptoms = 1; faculty affiliation and anxiety = 2; see also **Table 2** and **Supplementary Material S3**).

**Table 2:** Results of the multiple linear regression with the RDoC domain scores (PVS-, NVS-, CS- and SP-score) as predictors and stress, anxiety and depressive symptoms as criterions.

Outcome: stress symptoms							
Predictors	*b*	*SE*	*β*	*t*	*p*	95% *CI*	
						LB	UB
(intercept)	7.475	0.214		34.992	0.000	7.053	7.896
PVS-score	0.474	0.458	0.089	1.035	0.302	-0.430	1.379
NVS-score	3.417	0.511	0.634	6.690	< 0.001*	2.409	4.425
CS-score	1.412	0.401	0.267	3.517	0.001*	0.620	2.203
SP-score	0.052	0.608	0.009	0.085	0.933	-1.148	1.251
N = 182 (outliers = 2); R^2^ = 0.621; *R^2^_corr_* = 0.613; F(4, 177) = 72.628; p < 0.001							
Outcome: anxiety symptoms							
Predictors	*b*	*SE*	*β*	*t*	*p*	95% *CI*	
						LB	UB
(intercept)	4.717	0.179		26.402	0.000	4.364	5.069
PVS-score	0.671	0.383	0.152	1.755	0.081	-0.083	1.426
NVS-score	2.753	0.428	0.612	6.434	< 0.001*	1.909	3.598
CS-score	0.994	0.329	0.227	3.022	0.003*	0.345	1.644
SP-score	-0.484	0.508	-0.107	-0.953	0.342	-1.487	0.519
N = 182 (outliers = 2); R^2^ = 0.619; *R^2^_corr_* = 0.610; F(4, 177) = 71.876; p < 0.001							
Outcome: depressive symptoms							
Predictors	*b*	*SE*	*β*	*t*	*p*	95% *CI*	
						LB	UB
(intercept)	7.910	0.214		37.003	0.000	7.488	8.332
PVS-score	-1.306	0.469	-0.210	-2.786	0.006*	-2.232	-0.381
NVS-score	1.519	0.522	0.240	2.910	0.004*	0.489	2.549
CS-score	1.000	0.393	0.163	2.543	0.012*	0.224	1.776
SP-score	-2.050	0.628	-0.323	-3.264	0.001*	-3.289	-0.810

N = 183 (outliers = 1); R^2^ = 0.718; *R^2^_corr_* = 0.711; F(4, 178) = 113.076; p < 0.001

*b*, regression coefficient; *SE*, standard error; *β*, standardized coefficient; *t*, t-tests; *p*, significance; *95% CI*, 95% confidence interval; *LB*, lower bound; *UB*, upper bound; *PVS-score*, domain score positive valence system; *NVS-score*, domain score negative valence system; *CS-score*, domain score cognitive systems; *SP-score*, domain score social processes. Significant results (p ≤ 0.05) are marked with *.

Interpretation of the corrected determination coefficient (*R^2^_corr_*) was based on the criteria proposed by Cohen (53), according to whom the respective regression model with an *R^2^_corr_* ≥ 0.02 represents a small, ≥ 0.13 a medium, and ≥ 0.26 a high explanatory power.

Multicollinearity indicators for all three MLRs relating to the domain factor scores did not violate standard thresholds, with VIFs consistently below 10, and tolerance values remained above 0.1. However, VIFs deviated notably from 1 on average, and tolerance values for the SP-domain were below 0.2. This suggests there was some degree of intercorrelation among predictors, particularly in the SP-domain, indicating potential, but not critical, concerns regarding multicollinearity. These values warrant cautious interpretation of individual regression coefficients, but do not indicate a violation of assumptions. The MLRs examining anxiety and depressive symptoms also violated the criterion of homoscedasticity. Hence, a new regression analysis was conducted using the HC3-method (54). However, this did not lead to changes in significance of the predictors, therefore, we report the original results.

For the MLRs examining faculty affiliation, the faculty with the highest average values (the Faculty of Science, i.e. Mathematics and Natural Sciences) was selected as the reference category for all three outcomes under consideration. All three MLRs examining faculty affiliation and sex did not show normally distributed residuals. For the same predictors related to anxiety, two outliers were detected and excluded. Heteroscedasticity was present. Therefore, a parameter estimate with robust standard errors based on the HC3-method was calculated for comparison (54). However, this did not lead to changes in significance, hence we report the original results. The subanalysis was restricted to participants with sufficient cell sizes in the sex categories; individuals identifying as diverse (n = 1) or not specifying their sex (n = 3) were therefore excluded.

## 3 Results

### 3.1 Descriptive statistics of mental health outcomes and RDoC domain scores

Among the students, 28.8% showed scores above the respective cut-off values for stress, 31.0% for anxiety, and 30.4% for depressive symptoms. Male students were significantly more often affected by depressive symptoms above cut-off than female students (χ²(1, N = 180) = 5.96, *p* = 0.015; **Supplementary Material S2**). Regarding mental burden assessed by the DASS-21 questionnaire, the results show a mean score of 7.0 ± 4.6 for the stress subscale, a score of 4.3 ± 3.9 for the anxiety subscale, and a score of 7.0 ± 5.3 for the depressive symptoms subscale. For females, the observed scores for stress 7.3 ± 4.4 and anxiety 4.5 ± 4.1 were higher than for males (stress subscale: 5.8 ± 5.1; anxiety subscale: 3.6 ± 3.4) and lower for the depressive symptom subscale (6.7 ± 5.1 in females vs. 7.8 ± 5.8 in males).

Students in the Faculty of Science reported the highest average scores on the stress (7.8 ± 4.7 (M ± SD)), anxiety (4.9 ± 4.1), and depressive symptoms subscale (8.0 ± 5.6). A similar trend appeared in the domain scores, with the Faculty of Science displaying the lowest average PVS- (0.03 ± 0.82) and SP-scores (-0.00 ± 0.87), and the highest average NVS- (0.02 ± 0.90) and CS-scores (0.08 ± 0.93). Values for the DASS-21 subscales and domain factor scores by faculty affiliation are presented in **Table 1**. However, these differences in mental health burden between faculties were not statistically significant (see **Table 1**).

### 3.2 Association of RDoC domain scores and stress

The multiple linear regression model using the four domain scores (PVS, NVS, CS, and SP) as predictors and stress symptoms as the outcome variable yielded an adjusted *R^2^_corr_* = 61.3% (*F*(4, 177) = 72.628; *p* < 0.001), indicating a statistically significant model fit significant model with high explanatory power (**Table 2**).

Results of the regression analyses (**Table 2**) showed a significant, positive association between the NVS-score and stress (*b* = 3.417; *SE* = 0.511; *t* = 6.690; *p* < 0.001) and the CS-score and stress (*b* = 1.412; *SE* = 0.401; *t* = 3.517; *p* = 0.001). There was no significant association between the PVS-score and stress (*b* = 0.474; *SE* = 0.458; *t* = 1.035; *p* = 0.302), nor between the SP-score and stress (*b* = 0.052; *SE* = 0.608; *t* = 0.085; *p* = 0.933).

### 3.3 Association of RDoC domain scores and anxiety

The multiple linear regression model with the four domain scores (PVS, NVS, CS, and SP) as predictors and anxiety symptoms as the outcome variable yielded an adjusted *R^2^_corr_* = 61.0% (*F*(4, 177) = 71.876; *p* < 0.001), indicating a significant model with high explanatory power (**Table 2**).

There was a statistically significant positive association between the anxiety symptom score and both the NVS-score (*b* = 2.735; *SE* = 0.428; *t* = 6.434; *p* < 0.001) and the CS-score (*b* = 0.994; *SE* = 0.329; *t* = 3.022; *p* = 0.003). There was no significant association between the anxiety score and either the PVS-score (*b* = 0.671; *SE* = 0.383; *t* = 1.755; *p* = 0.081) or the SP-score (*b* = -0.484; *SE* = 0.508; *t* = -0.953; *p* = 0.342; **Table 2**).

### 3.4 Association of RDoC domain scores and depressive symptoms

The multiple linear regression model with the four domain scores (PVS, NVS, CS, and SP) as predictors and depressive symptoms as the outcome variable yielded an adjusted *R^2^_corr_* = 71.1% (*F*(4, 178) = 113.076; *p* < 0.001; **Table 2**), indicating a significant model fit with high explanatory power.

All four domain scores were significantly associated with the depressive symptom score. The PVS- and SP-score showed a negative association with depressive symptoms (PVS: *b* = -1.306; *SE* = 0.469; *t* = -2.786; *p* = 0.006; SP: *b* = -2.050; *SE* = 0.628; *t* = -3.264; *p* = 0.001). The NVS- and CS-score both showed a positive association with depressive symptoms (NVS: *b* = 1.519; *SE* = 0.522; *t* = 2.910; *p* = 0.004; CS: *b* = 1.000; *SE* = 0.393; *t* = 2.543; *p* = 0.012).

### 3.5 Faculty affiliation and stress, anxiety, and depressive symptoms

Faculty affiliation, controlling for sex, did not make a significant explanatory contribution regarding stress (*R^2^_corr_* = 0.004; *F*(6, 173) = 1.125; *p* = 0.350), anxiety (*R^2^_corr_* = 0.000; *F*(6, 171) = 1.011; *p* = 0.420) or depressive (*R^2^_corr_* = 0.003; *F*(6, 173 = 1.082; *p* = 0.375) symptom scores (**Supplementary Material S3**). Although the overall regression model did not reach statistical significance, a statistically significant difference in the prevalence of depressive symptoms was observed between students of the Faculty of Human Sciences and those of the Faculty of Science (*b* = -2.044, *SE* = 1.014; *t* = -2.015; *p* = 0.045, **Supplementary Material S3**).

## 4 Discussion

The aim of the present study was to investigate the associations between the RDoC PVS-, NVS-, CS-, and SP-domains and the mental burden of stress, anxiety, and depressive symptoms in a sample of university students. Based on previous findings that suggested differences in mental health and symptom burden across different academic fields of study, we also explored whether mental burden was associated with faculty affiliation.

### 4.1 Prevalence of mental burden

With 28.8% of participants experiencing stress, 31.0% anxiety, and 30.4% depressive symptoms, we found evidence for a notable mental burden in our university student sample.

Direct comparisons with previous work are only partially possible, as the cited studies employed stress measures other than the DASS-21 used in our research. Acknowledging this methodological caveat, the share of participants who exceeded the DASS-21 cut-off for at least moderate stress in our sample was roughly twice as high as estimates for the general German population aged 18–64 years (women 31.1% vs. 13.9%; men 22.2% vs. 8.2%) (2). Studies employing different instruments among university students report markedly divergent prevalence rates—16.1% (55), 25.3% (8), 44.0% (1) and 48.1% (25)—placing our findings in the mid-to-upper range of this spectrum. Consistent with earlier work, female students in our sample reported higher mean stress scores than male students (*p* = 0.031 (8, 25, 55); **Supplementary Material S2**). However, when DASS-21 cut-off values were compared instead of mean scores, this difference was no longer significant (*p* = 0.255) (**Supplementary Material S2**).

Direct comparisons with earlier work regarding anxiety symptoms must again be considered with caution, as most of the cited studies assessed anxiety and depressive symptoms with instruments other than the DASS-21 used in our study. Bearing this limitation in mind, 31.0% of our participants exceeded the DASS-21 cut-off for at least moderate anxiety - roughly twice the estimate for the German population aged 18–64 years (15.3%) (2). Relative to their population peers, female students were affected about 1.5-fold (29.6%), and male students nearly three-fold (33.3%). Student surveys using alternative measures have more frequently reported lower anxiety prevalences - 16.3% (24), 17.8–20.9% (56), 24.3% (55), 17.4% (8) - but occasionally higher ones as well (42.2% (57), 38.0% (25)).

A similar pattern emerged for depressive symptoms. Using DASS-21 thresholds, the proportion of students with at least moderate depressive symptoms in our sample clearly exceeded population estimates, with the gap most pronounced among men (women: 25.2% vs. 13.1%; men: 44.4% vs. 6.4%) (2). The majority of prior student studies - again based on different scales - reported lower rates (14.2% (2), 25.0% (57), 20.0–20.3% (56), 24.0% (55), 15.6% (8)), although a higher prevalence (35.1% (25)) has also been documented.

Beyond comparisons with German population norms, meta analytic evidence also indicates that elevated levels of anxiety and depressive symptoms are common in university and college students worldwide and fall within the range of observed prevalences in our sample. Li et al. (58) reported in their global meta analysis pooled prevalence estimates of approximately 39% for anxiety and 34% for depressive symptoms in college and university students, although rates varied across countries and subgroups (e.g., higher symptoms in lower middle income countries and medical students). Chang et al. (59) reported in their meta-analysis of college and university students during the COVID-19 pandemic pooled prevalence estimates of approximately 31% for anxiety symptoms and 34% for depressive symptoms, with higher rates of depressive symptoms observed in female students and variation across countries. Consistent with these findings for gender differences, previous research in Germany has also pointed to a higher burden of depressive and anxiety symptoms among female university students (24). Contrary, in our sample, male students were significantly more affected by depressive symptoms than females (men: 44.4%, women: 25.2%, *p* = 0.015) (**Supplementary Material S2**). From an RDoC perspective, the co-occurrence of stress, anxiety, and depressive symptoms in our sample is consistent with a framework of overlapping negative valence system processes, while also reflecting symptom-specific variations.

It is important to consider the small sample size and limited generalizability resulting from data collection at a single site in a cross-sectional design when interpreting these findings. Regional influences per se, however, are not likely to explain the results, as the studies we referred to have all been conducted among German students from different universities and cities (24, 25, 55, 56), except for the study by McIntyre et al. (57). Moreover, differences in employed measures limit comparability. An additional consideration may be that participation in a survey on mental health topics may have appealed to students experiencing psychological burden or those with a specific interest in mental health to a greater extent, therefore potentially introducing a self-selection bias. Beyond the specific associations between RDoC domains and mental health outcomes, the present findings need to be interpreted in light of the developmental and contextual characteristics of the sample. University students typically fall within the period of emerging adulthood, a life stage associated with increased vulnerability for the onset of mental disorders, heightened emotional reactivity, and ongoing neurocognitive development (23, 60, 61). University attendance often coincides with first-time exposure to academic demands, financial pressure, role transitions, and changes in social support networks (61). These factors may not only increase psychological distress but also interact with core RDoC domains, such as negative valence processes (e.g., heightened threat sensitivity), cognitive systems (e.g., perceived impairments under stress), and social processes (e.g., changes in affiliation and belonging). Consequently, the elevated prevalence of stress, anxiety, and depressive symptoms observed in our sample may in part be related to age-related vulnerability and contextual stressors inherent to the university environment.

### 4.2 Domain scores and stress

The positive relationship between the NVS-score and stress found in our study is consistent with previous findings that reported significant associations between stress and negative affectivity (62, 63). We also found a positive relationship between the CS-score and stress symptoms, which resonates with findings on the association of stress symptoms and impairments in cognitive functioning such as working memory performance, inhibited executive performance, and reduced cognitive flexibility (37, 64, 65). Notably, the NVS-score showed a stronger association with the level of stress than the CS-score.

In our study, we did not find an association between the PVS-score and stress, which falls in line with previous work by Berenbaum and Connelly (34), who did not find a relationship between hedonic capacity and subjective stress level. However, Admon et al. (66) demonstrated impaired responsiveness to reward following a highly stressful event using the example of a military deployment, which might suggest an association of the PVS-score and stress. The results may be explained by differences in subjectively perceived intensity and duration of the stress symptoms; Baik (67) reported, that the duration of stress exposure influenced the reward system. Short-term stress may cause promotion of reward-related processes in the brain, while prolonged stress could lead to inhibition (67). Since it is not entirely clear whether the stress subscale of the DASS-21 used in our study assesses acute or chronic stress (51), this might influence the results due to opposing associations.

In contrast to our findings of no association between SP and stress, Sinnaeve et al. (39) showed that chronic stress had a negative impact on all social processes considered in the RDoC framework. Notably, the items on the DASS-21-stress subscale do not explicitly refer to social situations, which leaves room for interpretation and may contribute to the differences in findings.

### 4.3 Domain scores and anxiety

The positive relationship between the NVS-score and the severity of anxiety symptoms in our sample is consistent with previous findings by Förstner et al. (40), who reported positive associations between NVS and the severity of anxiety. This aligns with findings of sensitivity to acute and potential threat stimuli in connection with pathological anxiety (e.g., for a synthesis see (68)).

Our findings of a positive association between anxiety and the CS-score fall in line with previous research on the association between cognitive impairments and anxiety. Ferreri et al. (41) and Castaneda et al. (42) reported a link between increased anxiety symptoms and impaired executive cognitive performance.

In our study, we did not find an association of the PVS-score with anxiety symptoms. Even though Förstner et al. (40) found such an association, they also noted that the association between the PVS-score and anxiety symptoms was weaker than the association between the PVS-score and depressive symptoms. Additionally, neither the severity of anxiety nor depressive symptoms could be interpreted as significantly increased based on the DASS-21 cut-off value in our sample. The study by Förstner et al. (40) was based on a clinical sample of patients with anxiety disorders or depression, which on average showed considerably lower PVS-scores compared to our student sample. Our results fall in line with Medeiros et al. (69) findings, who also did not find associations between anxiety symptoms and the PVS.

Contrary to our findings of no relationship between SP and anxiety symptoms, Saris et al. (70) and Förstner et al. (40) reported significant, negative associations between social functioning and anxiety disorders. It is to note, that Förstner et al. (40) and Saris et al. (70) found stronger associations between SP and depression than anxiety. Additionally, the items on the DASS-21 anxiety subscale primarily refer to the experience of physical anxiety symptoms and includes only one item referring to situational anxiety. Finally, the SP-score in our sample was 0.17 on average, compared to -0.30 in the clinical sample of Förstner et al. (40), suggesting that social processes were less impaired in our participants, which may explain a lack of significance in our study.

### 4.4 Domain scores and depressive symptoms

Förstner et al. (40) also found a positive association between the NVS-score and depressive symptoms, in line with our results. Jaworska et al. (71) pointed out that depressive symptoms were associated with increased activation of brain areas in detection of negative (visual) stimuli and processing of negative emotions.

Our findings of a positive association between the CS-score and depressive symptoms are in line with findings showing impaired cognitive functioning in patients with a first depressive episode compared to healthy controls (72). For example studies reported impaired executive neural performance, particularly working memory, cognitive flexibility, planning ability, and cognitive control (42, 46, 73).

Our findings align with studies showing low positive affect and impaired sensitivity, processing and responsiveness to reward stimuli in association with depressive symptoms (44, 74, 75). Impaired responsiveness to reward stimuli was also observed in a non-clinical sample of undergraduate students comparable to the present sample (76). In their review, Carcone and Ruocco (77) point out that an impairment of the PVS was not only associated with diagnosed depression but was also found in individuals at increased risk due to family history.

Consistent with our findings, Förstner et al. (40) and Saris et al. (70) found a negative association between the SP-score and depressive symptoms. This observation parallels findings from research showing impaired social functioning (e.g. social anhedonia, increased sensitivity to social exclusion, difficulties in maintaining social relationships) in people diagnosed with depression (78).

### 4.5 Faculty affiliation in association with stress, anxiety, and depressive symptoms

Contrary to our hypothesis, we did not find an association between faculty affiliation and the level of stress, anxiety, and depressive symptoms. Sex also did not show a significant association with the outcomes. Descriptively however, we found variations in symptom levels between faculties, with students from the Faculty of Science reporting a higher average burden across all three outcomes – an observation that is consistent with previous research (25, 56). Importantly though, the differences were not statistically significant which is in contrast to findings reporting significant variations in psychological distress depending on the field of study (25, 56) and only aligns with results reported by Grützmacher et al. (8) who found no significant differences depending on the field of study in relation to the experience of stress. Regarding prevalence of depressive symptoms, the Faculty of Human Science reported a significantly lower burden compared to the Faculty of Science (*p* = 0.045) (**Supplementary Material S3**). Since the overall model was not significant, this conclusion must be interpreted with caution.

The small sample size and representation of individual faculties in particular (e.g. Law Faculty (n = 11), Digital Engineering Faculty (n = 13)) might influence the results, compared to findings in lager sample sizes (e.g. Grützmacher et al. (8), who surveyed a total of 6198 students, and Blaszcyk et al. (25), who surveyed 2826 students).

### 4.6 Limitations and strengths

This study has several limitations. Firstly, our relatively small sample size and data collection at one study site only limits comparability and generalizability of the results. Secondly, this study relied on self-report only. Hence, interpretation of our findings regarding the CS-score in particular may be limited, as only the subjective perception of participants’ cognitive functioning was recorded, while objective assessments may be able to provide further insights into cognitive functioning. Moreover, we only included four domains of the RDoC matrix in the present analyses, and we analyzed data on domain level only. Including all domains of the framework as well as analyses on (sub-)construct level may capture psychological functioning more comprehensively and provide further insights into underlying mechanisms. Further, the RDoC framework also stresses the influences of the external environment and development, which should be given more consideration in future research.

Regarding the statistical analyses, the finding of high multicollinearity of the domain scores might influence the results, even though there was no clear violation of the assumption. High multicollinearity could be expected given the nature of the domain scores, as clear differentiation has been difficult, and they may partially overlap or interact (79). Nevertheless, increased standard errors potentially limiting comparability of the strengths of association between the respective predictors and the outcome (80) should be taken into account when interpreting the effect sizes (*β*). Moreover, multicollinearity can result in significant correlations being falsely reported as non-significant (81).

Nevertheless, the study also presents strengths. The employed DASS-21 is a psychometrically valid, non-diagnostic, dimensional instrument well suited for assessing stress, anxiety, and depressive symptoms in non-clinical populations, as it was originally developed and validated largely in such samples. Its use in the present study enabled the assessment of multiple symptom domains within a university student population and provided useful insights into the extent and profile of mental burden across different faculties. Moreover, by conceptualizing symptoms as continuous dimensions, the study contributes to a transdiagnostic perspective within the RDoC framework. The observation that similar RDoC-relevant systems were associated with multiple symptom domains, alongside a substantial proportion of students reporting elevated symptom levels, may inform understanding of vulnerability processes underlying different manifestations of distress. Moreover, our study represents a further step in conceptualizing and understanding mechanisms of stress, anxiety, and depressive symptoms based on the RDoC framework and how this approach can be employed when investigating health in a non-clinical university student sample.

## 5 Conclusion

In summary, our findings indicate significant positive associations between the NVS- and CS-score and the burden of stress, anxiety, and depressive symptoms. Mental burden regarding all three outcomes was therefore reflected in heightened negative affect, anxiety, and (subjectively) impaired cognitive functioning. For depressive symptoms, we found significant negative associations with the PVS- and SP-score, reflecting reduced positive affect and impaired social behavior and interaction. We did not find evidence for faculty-specific burden; thus, no faculty specific recommendations can be made based on our results. Nevertheless, approximately 30% of university students in our sample showed significant psychological distress, highlighting the urgent need for expanded, tailored prevention programs and support services for this particular population. Further research should aim to include additional units of analyses using not only self-report but also external validators to assess key variables and expand assessments to include all (current) RDoC domains and different units of analysis. Longitudinal studies may be particularly useful for identifying critical (study) phases associated with mental burden and for providing information on the directionality of observed relationships. A deeper understanding of the underlying characteristics of stress, anxiety, and depressive symptoms in the PVS, NVS, CS and SP has the potential to provide critical insights into and can inform the development of needs-based, early intervention and prevention strategies in university student populations.

## Data Availability

The raw data supporting the conclusions of this article will be made available by the authors, without undue reservation.
