# Supplementary material for: The Research Domain Criteria (RDoC) domains positive valence system, negative valence system, cognitive systems, and social processes and their relationship with stress, anxiety, and depressive symptoms in a university student sample
Source: Front Psychiatry. 2026 Mar 5;17:1674802. doi: 10.3389/fpsyt.2026.1674802 (PMC12999574; doi:10.3389/fpsyt.2026.1674802)
Supplement: Supplementary file 1 [file Supplementaryfile1.docx]

**Supplementary Material S1:
Confirmatory Factor Analysis (CFA) for RDoC Domain factor scores:**

**Methods:**

The selection of variables was informed by a comprehensive questionnaire battery developed through a Delphi consensus process, incorporating expertise from both DZPG-affiliated researchers and international collaborators (see OSF <https://osf.io/ec9hs/>; Tschorn et al., 2025). The included items utilized diverse response formats, including Likert-type scales, bubble ratings, and visual analogue scales (VAS). In the bubble rating format, participants evaluated clusters of semantically related descriptors, visually grouped as a single unit (‘bubble’), by assigning a global rating on a Likert scale that captured their overall impression of the cluster. All items were presented in German and were administered across multiple digital platforms, including smartphones, tablets, and laptops.

The latent RDoC domain structure on which the MDS Valid instrument is based was determined using a confirmatory factor analysis (CFA) with ML estimator on item level and full information maximum likelihood (FIML) handling missings. It was executed in a manner consistent with the approach employed by Foerstner et al. (2023). For the statistical calculation, both the data of the UP students (n = 249, 41.8%) and those of the participants recruited on the basis of the healthcare centers belonging to the DZPG (n = 347, 58.2%) were used in order to obtain a sufficiently large sample.

The initial model was structured as follows (all variables were centered prior to analysis):

| **Factor** | **Variable** |
| --- | --- |
| **PVS:** Positive Valence Systems | - DSM5CC_1 - PANAS_active, PANAS_interested, PANAS_excited, PANAS_determined |
| **NVS:** Negative Valence Systems | - vas_SUDS - DSM5CC_6, DSM5CC_7, DSM5CC_8a - PID5_6 - PANAS_ashamed, PANAS_irritable |
| **CS:** Cognitive Systems | - DSM5CC_15 - BRIEFA_a1, BRIEFA_a2, BRIEFA_a3 |
| **SP:** Social Processes | - WHODAS20_11r - DSM5CC_1a - vas_social_exclusion - vas_social_discrimination - social_support - social_support_bubble rating - IPSM_24, IPSM_30, IPSM_33, IPSM_36 - LPFSBF20_7 - DSM5CC_3 - ERQ_7, ERQ_8, ERQ_2, ERQ_6 - LHA_AGG_4 |
| **ARS: Arousal and Regulatory Systems** | - DSM5CC_14 - POMS_vigorous - POMS_exhausted - POMS_fatigued - POMS_worn out |
| **SMS: Sensorimotor Systems** | - AES_3_st - AES_17 - AES_2 - AES_10 - AES_16 |

Note. AES = Apathy Evaluation Scale, BRIEF = altered version based on the Behavior Rating Inventory of Executive Function, bubble rating = graphical rating of perceived social support, DSM5CC = CCSM DSM-5 Level 1 Cross-Cutting Symptom Measure, ERQ = Emotion Regulation Questionnaire, IPSM = Interpersonal Sensitivity Measure, LHA = Life History of Aggression, LPFS-BF 2.0 = Level of Personality Functioning Scale – Brief Form (20 items), PANAS = Positive and Negative Affect Scale, PID-5 = Personality Inventory for DSM-5, POMS = Profile of Mood States, social support = perceived social support (single-item measure), SUDS = Subjective Units of Distress Scale, vas = Visual Analogue Scale, WHO-DAS 2.0 = WHO Disability Assessment Schedule 2.0.

Since the initial model had insufficient fit indices (CFI=.717, TLI=.695, RMSEA=.092, SRMR=.078), the following procedure was applied:

First, variables with R² < .20 were systematically removed, resulting in the exclusion of 11 items (PID5_64, Social_Discrimination, IPSM_30, IPSM_33, IPSM_36, LPFSBF20_7, ERQ_7, ERQ_8, ERQ_2, ERQ_6, LHA_AGG_4, R² see below) from this step in the revised model.

Variables with R² < .20

| **Variable** | **Estimate** |
| --- | --- |
| PID5_64 | 0.140 |
| Social_Discrimination | 0.060 |
| IPSM_30_ | 0.184 |
| IPSM_33 | 0.095 |
| IPSM_36 | 0..84 |
| LPFSBF20_7 | 0.160 |
| ERQ_7 | 0.131 |
| ERQ_8 | 0.071 |
| ERQ_2 | 0.116 |
| ERQ_6 | 0.081 |
| LHA_AGG_4 | 0.002 |

Subsequently, adjustments were also made based on modification indices > 50, with theoretical plausibility guiding decisions. All relevant modification indices and the corresponding decisions are described below.

| **Modification** | **Modification Index (MI)** | **Expected Parameter Change (EPC)** | **Standardized Estimate (sepc.all)** | **Interpretation, decision** |
| --- | --- | --- | --- | --- |
| IPSM_30 ~~ IPSM_36 | 194.36 | 0.559 | 0.652 | High residual correlation – potentially redundant items, IPSM_3 removed |
| ERQ_2 ~~ ERQ_6 | 168.21 | 0.563 | 0.625 | Items from the same scale – ERQ_6 removed |
| ERQ_7 ~~ ERQ_8 | 145.27 | 0.522 | 0.582 | Conceptually similar items – ERQ_8 removed |
| DSM5CC_1 ~~ DSM5CC_1a | 114.78 | 0.233 | 0.584 | Likely overlapping content - DSM5CC_1 removed |
| PANAS_irritable~~ DSM5CC_3 | 106.11 | 0.350 | 0.495 | Cross-scale residual correlation – may reflect conceptual or methodological overlap - DSM5CC_3 removed |
| SoP loads on DSM5CC_1 | 104.50 | –0.785 | –0.781 | Strong negative cross-loading – DSM5CC_1 removed |

Nevertheless, the six-domain CFA model continued to show insufficient global fit (CFI = .89). From a theoretical perspective, this may reflect both the substantial conceptual overlap among RDoC domains at higher levels of abstraction and the incremental expansion of the RDoC framework over time. We therefore evaluated a theory-driven domain reduction by excluding the Arousal/Regulatory Systems (ARS) and Sensorimotor Systems (SMS) domains, which were introduced in later stages of the RDoC development process. This reduction effectively reoriented the model toward an earlier version of the RDoC matrix, as also applied in Foerstner et al. (2023). The resulting four-domain model demonstrated an acceptable improvement in model fit (CFI = .91). It should further be noted that the comparatively low fit of the initial model may, at least in part, be attributable to the use of a maximum likelihood estimator, which is known to be sensitive to deviations from multivariate normality and complex item distributions.

The following figure shows the final CFA model and the corresponding factor loadings resulting in our four domain factorscores used for the regression models in this study. The comparative fit index for the final model was CFI=0.91, the Tucker-Lewix-Index was TLI=.89 (RMSEA=.074; SRMR=.054), indicating a sufficient fit for the collected data (Hu & Bentler, 1999).

*Factor loadings of the RDoC domains with the z-standardized latent domain scores PVS, NVS, CS, and SP and the questionnaire items used, respectively*

**

*Note*. Latent variables: *SP* social processes, *PVS* positive valence system, *NVS* negative valence system, *CS* cognitive systems. Observed variables: *WHO-DAS 2.0 friendships* World Health Organization Disability Assessment Schedule 2. 0 Item 11 - Friendships, *DSM5CC 1 social anhedonia* DSM-5 Self-Rated Level 1 Cross-Cutting Symptom Measure - Adult Item 1a Social Anhedonia adapted, *VAS social exclusion* Visual Analogue Scale Social Exclusion, *SOZU* s*ocial support* KiGGS study (Robert Koch-Institute) item on social support, *b_SOZU social support* Bubble Rating and Visual Analogue Scale Social Support, *IPSM social contact* Interpersonal Sensitivity Measure Item 24 - Uncertainty in Social Contact, *PANAS active* PANAS item “active”, *PANAS interested* PANAS item “interested”, *PANAS excited* PANAS item “excited”, *PANAS determined* PANAS item “determined”, *VAS acute threat* visual analogue scale “subjective units of distress” (SUDS), *DSM5CC6 anxiousness* DSM5CC item 6 anxiousness, *DSM5CC 7 anxiousness* DSM5CC item 7 anxiousness, *DSM5CC 8 phobic anxiety* DSM5CC item 8 phobic anxiety adapted, *PANAS ashamed* PANAS item “ashamed”, *PANAS irritable* PANAS item “irritable”, *DSM5CC 15 (working)memory* DSM5CC item 15 executive functions (working)memory, *BRIEFA 1 (working)memory* Behaviour Rating Inventory of Executive Function item 1 executive functions - (working)memory adapted, *BRIEFA 2 flexibility* BRIEF item 2 executive functions - flexibility adapted, *BRIEFA 3 planning* BRIEF item 3 executive functions - planning adapted
For all references, see: OSF <https://osf.io/ec9hs/>; Tschorn et al., 2025

The following table shows all factor loadings for the final model:

| **Latent Factor** | **Indicator** | **Unstandardized Estimate (B)** | **Standard Error (SE)** | **Z-Value** | **Standardized Estimate (Beta)** | **Significance** |
| --- | --- | --- | --- | --- | --- | --- |
| **PVS** | PANAS_active | .748 | .041 | 18.093 | .746 | *** |
| **PVS** | PANAS_interested | .819 | .039 | 20.771 | .821 | *** |
| **PVS** | PANAS_excitedt | .822 | .040 | 20.757 | .822 | *** |
| **PVS** | PANAS_determined | .711 | .042 | 16.831 | .708 | *** |
| **NVS** | vas_SUDS | .678 | .042 | 16.185 | .684 | *** |
| **NVS** | DSM5CC_6 | .790 | .040 | 19.838 | .794 | *** |
| **NVS** | DSM5CC_7 | .801 | .040 | 20.195 | .804 | *** |
| **NVS** | DSM5CC_8a | .720 | .041 | 17.625 | .730 | *** |
| **NVS** | PANAS_ashamed | .669 | .043 | 15.621 | .667 | *** |
| **NVS** | PANAS_irritable | .446 | .046 | 9.711 | .448 | *** |
| **CoS** | DSM5CC_15 | .721 | .043 | 16.722 | .725 | *** |
| **CoS** | BRIEFA_a1 | .704 | .043 | 16.315 | .706 | *** |
| **CoS** | BRIEFA_a2 | .738 | .042 | 17.382 | .739 | *** |
| **CoS** | BRIEFA_a3 | .757 | .042 | 17.913 | .758 | *** |
| **SoP** | WHODAS20_11r | .729 | .042 | 17.267 | .728 | *** |
| **SoP** | DSM5CC_1a | -.741 | .042 | -17.739 | -.747 | *** |
| **SoP** | vas_SOZEX100 | -.728 | .042 | -17.206 | -.725 | *** |
| **SoP** | SOZU | .519 | .046 | 11.246 | .519 | *** |
| **SoP** | b_SOZU | .610 | .045 | 13.572 | .610 | *** |
| **SoP** | IPSM_24 | -.521 | .045 | -11.52 | -.524 | *** |

The RDoC domain factor scores for the latent constructs NVS, PVS, CS and SP were estimated from the final model.

The characteristics of the RDoC domain factor scores determined can be interpreted as follows: A low PVS score indicates a low level of hedonistic affect and is therefore associated with an impairment in the processing of positive stimuli and rewards. A high NVS score is associated with, among other things, more negative affectivity, a higher sense of stress and, anxiety. A high CS score is associated with self-reported problems and impairment of executive cognitive processes such as working memory, flexibility and planning ability. A low SP score reflects an impairment of social behavior and is reflected, among other things, in challenges in dealing with and more negative reactions to others.

References:

Tschorn, M., Böttger, S. J., Heinen-Stach, D., Foerstner, B. R., & Rapp, M. A. (2025, June 18). DZPG Minimum Data Set (MDS). <https://doi.org/10.17605/OSF.IO/EC9HS>

Foerstner, B. R., Tschorn, M., Reinoso‑Schiller, N., Mascarell Maričić, L., Röcher, E., Kalman, J. L., Stroth, S., Mayer, A. V., Schwarz, K., Kaiser, A., Pfennig, A., Manook, A., Ising, M., Heinig, I., Pittig, A., Heinz, A., Mathiak, K., Schulze, T. G., Schneider, F., ... Rapp, M. A. (2023). Mapping Research Domain Criteria using a transdiagnostic mini‑RDoC assessment in mental disorders: A confirmatory factor analysis. *European Archives of Psychiatry and Clinical Neuroscience, 273*(3), 527–539. https://doi.org/10.1007/s00406-022-01440-6

Hu, L. T., & Bentler, P. M. (1999). Cutoff criteria for fit indexes in covariance structure analysis: Conventional criteria versus new alternatives. *Structural Equation Modeling: A Multidisciplinary Journal, 6*(1), 1–55. https://doi.org/10.1080/10705519909540118
