# Supplementary material for: The Research Domain Criteria (RDoC) domains positive valence system, negative valence system, cognitive systems, and social processes and their relationship with stress, anxiety, and depressive symptoms in a university student sample
Source: Front Psychiatry. 2026 Mar 5;17:1674802. doi: 10.3389/fpsyt.2026.1674802 (PMC12999574; doi:10.3389/fpsyt.2026.1674802)
Supplement: Supplementary file 2 [file Supplementaryfile2.docx]

**Supplementary Material S2**

*Group differences between male and female students with regard to the DASS-21 (DASS-stress, DASS-anxiety and DASS-depression) and domain scores (PVS, NVS, CS and SP).*

|  | **group** | | |  |  |  |
| --- | --- | --- | --- | --- | --- | --- |
| **variable** | **overall sample**  **(*M* ± *SD*)** | **male**  **(*M* ± *SD*)** | **female**  **(*M* ± *SD*)** | ***p*** |  |  |
| **N (%)** | 180 (100.0) | 45 (25.0) | 135 (75.0) |  |  |  |
| **DASS-21** | | | | |  |  |
| DASS-stress | 7.0 ± 4.6 | 5.8 ± 5.1 | 7.3 ± 4.4 | 0.031* |  |  |
| DASS-anxiety | 4.3 ± 3.9 | 3.6 ± 3.4 | 4.5 ± 4.1 | 0.216 |  |  |
| DASS-depression | 7.0 ± 5.3 | 7.8 ± 5.8 | 6.7 ± 5.1 | 0.312 |  |  |
| **DASS-21 scores above cut-off in %** | | | | | | |
| DASS-stress | | 28.8 | 22.2 | 31.1 | 0.255 | |
| DASS-anxiety | | 31.0 | 33.3 | 29.6 | 0.640 | |
| DASS-depression | | 30.4 | 44.4 | 25.2 | 0.015* | |

*Note. M* mean; *SD* standard deviation; *p* significance; *DASS-21* Depression-Anxiety-Stress Scale; *DASS-stress* Depression-Anxiety-Stress Scale subscale stress; *DASS-anxiety* Depression-Anxiety-Stress Scale subscale anxiety; *DASS-depression* Depression-Anxiety-Stress Scale subscale depressive symptoms; *PVS-score* domain score positive valence system; *NVS-score* domain score negative valence system; *CS-score* domain score cognitive systems; *SP-score* domain score social processes; * significant at *p* < .05.
The Mann-Whitney U-test (*p* < 0.05) was used to statistically analyze group differences between male and female students.
