# Supplementary material for: The Research Domain Criteria (RDoC) domains positive valence system, negative valence system, cognitive systems, and social processes and their relationship with stress, anxiety, and depressive symptoms in a university student sample
Source: Front Psychiatry. 2026 Mar 5;17:1674802. doi: 10.3389/fpsyt.2026.1674802 (PMC12999574; doi:10.3389/fpsyt.2026.1674802)
Supplement: Supplementary file 3 [file Supplementaryfile3.docx]

**Supplementary Material S3**

*Results of the multiple linear regression with faculty affiliation and sex as predictors and stress, anxiety and depressive symptoms as criteria*

|  | **Outcome: stress symptoms** | | | | | | | | | | | | | | | | |
| --- | --- | --- | --- | --- | --- | --- | --- | --- | --- | --- | --- | --- | --- | --- | --- | --- | --- |
|  |  | |  | | | |  | |  | |  | | **95% *CI*** | | | | |
|  | **predictors** | | ***b*** | | ***SE*** | | ***β*** | | ***t*** | | ***p*** | | **LB** | | **UB** | | |
|  | (intercept) | | 6.658 | | 0.875 | |  | | 7.605 | | 0.000 | | 4.930 | | 8.386 | | |
|  | Law Faculty | | -1.657 | | 1.540 | | -0.086 | | -1.076 | | 0.283 | | -4.697 | | 1.383 | | |
|  | Faculty of Arts (interdisciplinary cultural studies) | | -1.297 | | 1.145 | | -0.094 | | -1.133 | | 0.259 | | -3.558 | | 0.963 | | |
|  | Faculty of Human Sciences | | -0.973 | | 0.895 | | -0.095 | | -1.086 | | 0.279 | | -2.740 | | 0.795 | | |
|  | Faculty of Economics and Social Science | | -0.828 | | 1.116 | | -0.062 | | -0.742 | | 0.459 | | -3.031 | | 1.375 | | |
|  | Digital Engineering Faculty | | -1.944 | | 1.445 | | -0.109 | | -1.345 | | 0.180 | | -4.797 | | 0.908 | | |
|  | sex | | 1.454 | | 0.827 | | 0.136 | | 1.757 | | 0.081 | | -0.179 | | 3.087 | | |
|  | *Note*. *b* regression coefficient; *SE* standard error; *β* standardized coefficient; *t* t-tests; *p* significance; * significant at *p* < .05; *95 % CI* 95 % confidence interval; LB lower bound; UB upper bound.  N = 180; *R^2^* = 0.038; *R^2^_corr_* = 0.004; F(6, 173) = 1.125; *p* = 0.350; reference category = Faculty of Science (Mathematics and Natural Sciences) | | | | | | | | | | | | | | | | |
|  | **Outcome: anxiety symptoms** | | | | | | | | | | | | | | | | |
|  |  | |  | |  | |  | |  | |  | | **95% *CI*** | | | | |
|  | **predictors** | | ***b*** | | ***SE*** | | ***β*** | | ***t*** | | ***p*** | | **LB** | | **UB** | | |
|  | (intercept) | | 3.964 | | 0.704 | |  | | 5.630 | | 0.000 | | 2.574 | | 5.354 | | |
|  | Law Faculty | | -2.099 | | 1.238 | | -0.136 | | -1.695 | | 0.092 | | -4.542 | | 0.345 | | |
|  | Faculty of Arts (interdisciplinary cultural studies) | | -0.581 | | 0.921 | | -0.053 | | -0.631 | | 0.529 | | -2.400 | | 1.237 | | |
|  | Faculty of Human Sciences | | -1.165 | | 0.725 | | -0.141 | | -1.607 | | 0.110 | | -2.597 | | 0.266 | | |
|  | Faculty of Economics and Social Science | | -0.014 | | 0.898 | | -0.001 | | -0.015 | | 0.988 | | -1.786 | | 1.758 | | |
|  | Digital Engineering Faculty | | -0.327 | | 1.161 | | -0.023 | | -0.281 | | 0.779 | | -2.619 | | 1.965 | | |
|  | sex | | 0.953 | | 0.665 | | 0.112 | | 1.433 | | 0.154 | | -0.360 | | 2.265 | | |
|  | *Note*. *b* regression coefficient; *SE* standard error; *β* standardized coefficient; *t* t-tests; *p* significance; * significant at *p* < .05; *95 % CI* 95 % confidence interval; LB lower bound; UB upper bound  N = 178 (outliers = 2); *R^2^* = 0.034; *R^2^_corr_* = 0.000; F(6, 171) = 1.011; *p* = 0.420; reference category = Faculty of Science (Mathematics and Natural Sciences) | | | | | | | | | | | | | | | | |
| **Outcome: depressive symptoms** | | | | | | | | | | | | | | | |  |  |
|  | |  | |  | |  | |  | |  | | **95% *CI*** | | | | |  |
| **predictors** | | ***b*** | | ***SE*** | | ***β*** | | ***t*** | | ***p*** | | **LB** | | **UB** | | |  |
| (intercept) | | 8.801 | | 0.992 | |  | | 8.872 | | 0.000 | | 6.843 | | 10.759 | | |  |
| Law Faculty | | -0.762 | | 1.745 | | -0.035 | | -0.437 | | 0.663 | | -4.207 | | 2.682 | | |  |
| Faculty of Arts (interdisciplinary cultural studies) | | -0.859 | | 1.298 | | -0.055 | | -0.662 | | 0.509 | | -3.421 | | 1.702 | | |  |
| Faculty of Human Sciences | | -2.044 | | 1.014 | | -0.176 | | -2.015 | | 0.045* | | -4.046 | | -0.041 | | |  |
| Faculty of Economics and Social Science | | -1.856 | | 1.265 | | -0.122 | | -1.468 | | 0.144 | | -4.352 | | 0.640 | | |  |
| Digital Engineering Faculty | | -0.399 | | 1.638 | | -0.020 | | -0.243 | | 0.808 | | -3.631 | | 2.834 | | |  |
| sex | | -1.039 | | 0.937 | | -0.086 | | -1.108 | | 0.269 | | -2.889 | | 0.812 | | |  |
|  | *Note*. *b* regression coefficient; *SE* standard error; *β* standardized coefficient; *t* t-tests; *p* significance; * significant at *p* < .05; *95 % CI* 95 % confidence interval; LB lower bound; UB upper bound  N = 180; *R^2^* = 0.036; *R^2^_corr_* = 0.003; F(6, 173) = 1.082; p = 0.375; reference category = Faculty of Science (Mathematics and Natural Sciences) | | | | | | | | | | | | | | | | |
